# Supplementary material for: Interplay between alkali-metal cations and silanol sites in nanosized CHA zeolite and implications for CO2 adsorption
Source: Commun Chem. 2023 Jun 29;6:134. doi: 10.1038/s42004-023-00918-1 (PMC10310731; doi:10.1038/s42004-023-00918-1)
Supplement: Supplementary file 2 — Supplementary Information [file 42004_2023_918_MOESM2_ESM.pdf]

Supporting information

Interplay between alkali-metal cations and silanol sites in nanosized CHA zeolite and implications for CO_2 adsorption

Sajjad Ghojavand[a], Eddy Dib[a], Jérôme Rey[b], Ayoub Daouli[b], Edwin B. Clatworthy[a], Philippe Bazin[a], Valérie Ruaux[a], Michael Badawi[b], Svetlana Mintova[a*]

[a] Normandie Université, ENSICAEN, UNICAEN, CNRS, Laboratoire Catalyse et Spectrochimie (LCS), 14000 Caen, France

[b] Université de Lorraine, CNRS, Laboratoire de Physique et Chimie Théoriques (LPCT), F-54000 Nancy, France

[*]Corresponding author. E-mail address: svetlana.mintova@ensicaen.fr, Tel: +33231452737

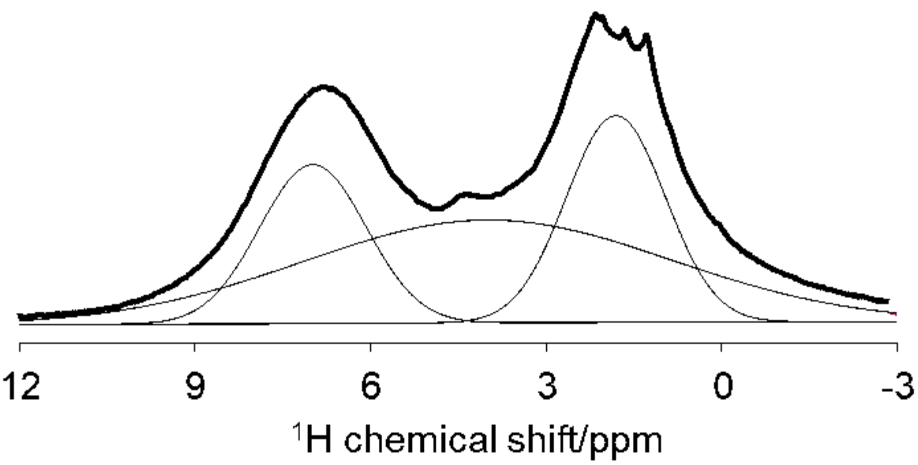

Fig. S1. ^{1}H MAS NMR signal of the empty 1.9 mm rotor rotating at 40 kHz. The three peaks used in the deconvolution are the ones used as a background for the deconvolution of all the ^{1}H NMR spectra.

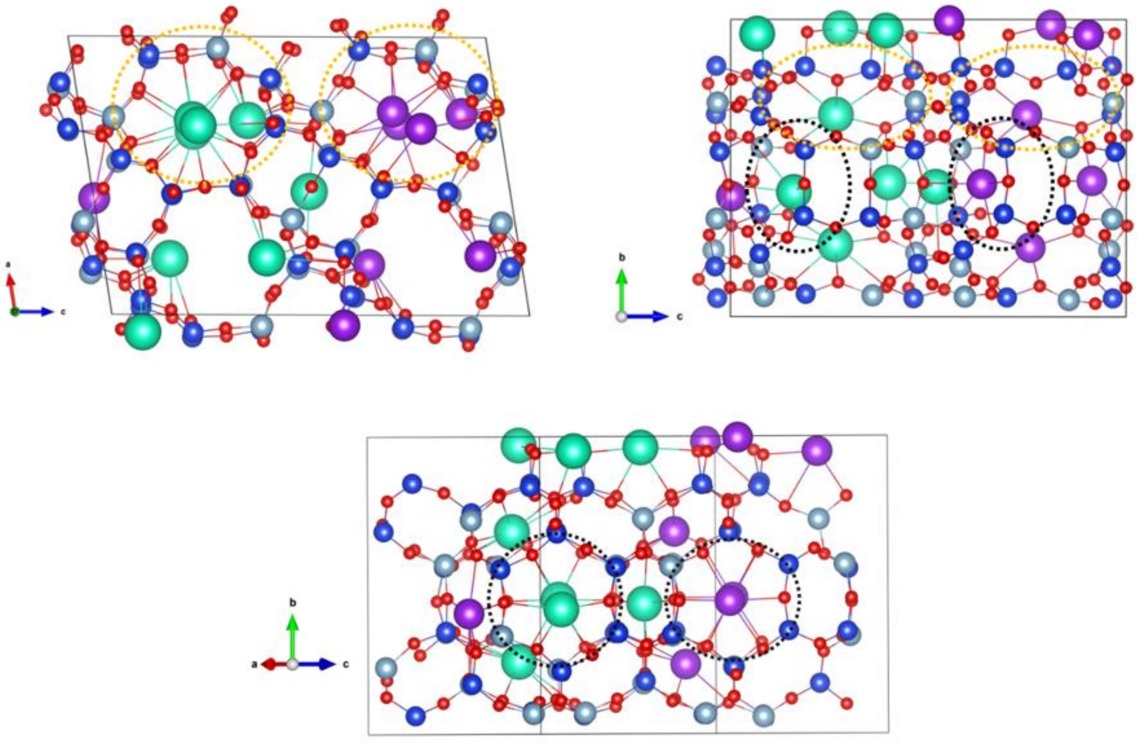

Fig. S2. Supercell of CHA used in the DFT simulations depicted in different planes. 8MR (cation site SIII’) is highlighted by orange circles and *d6r* (cation site SII) by black circles. Color code: Si in blue, O in red, Al in grey, K in purple, and Cs in green.

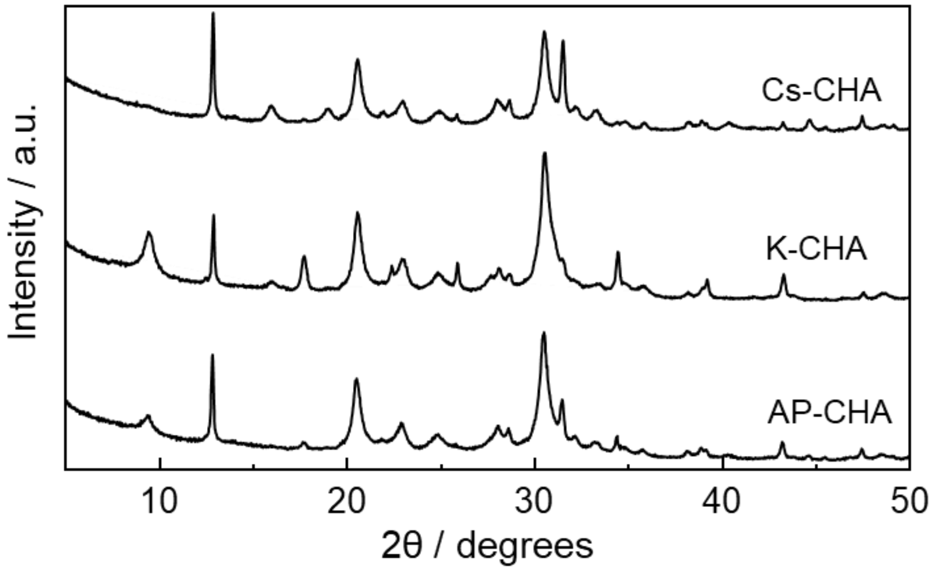

Fig. S3. XRD patterns of the nanosized AP-CHA, K-CHA, and Cs-CHA zeolite samples.

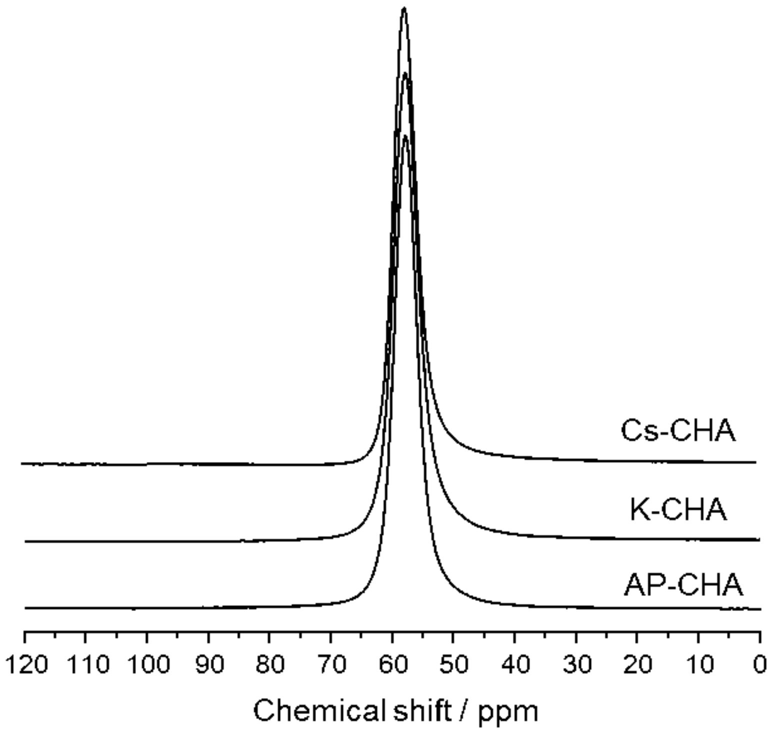

Fig. S4. ^{27}Al MAS NMR spectra of nanosized AP-CHA, K-CHA, and Cs-CHA CHA samples.

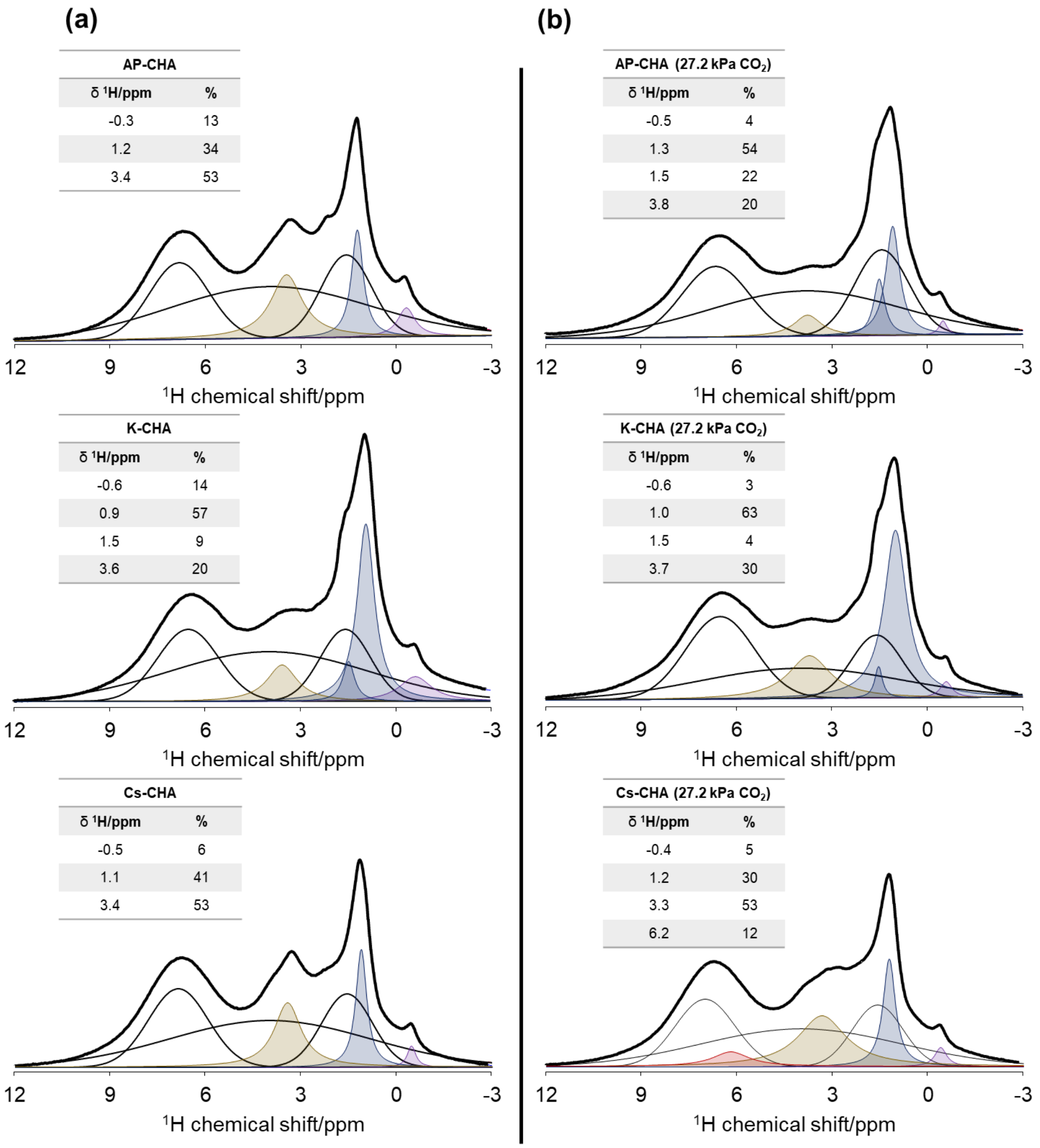

Fig. S5. ^{1}H MAS NMR spectra of nano-sized AP-CHA, K-CHA, and Cs-CHA zeolite samples (a) before and (b) after CO_2 adsorption (27.2 kPa); all samples were pretreated at 623 K under high vacuum (10^{-6} kPa) prior measurements. *Color code:* rotor signals in hollow black lines, purple for silanol sites interacting with cations, blue for isolated and weakly hydrogen bonded, yellow for moderately hydrogen bonded, and red for strong hydrogen bonded silanol sites. The shape of the rotor signal, varying because of the slight differences in magnetic susceptibility in the samples and it is kept at constant intensity during the fitting procedure for all samples.

Table S1. Electronic energies of adsorption ΔE_{ads} (in kJ mol^{-1}) of CO_2 in K- and Cs-CHA in the large cavity of the chabazite zeolite, with silanols in (a) a 6MR, (b) the confined corner of an 8MR next to a 4MR, and (c) an 8MR. The dispersion contribution ΔE_{disp} (in kJ mol^{-1}) is also reported. The calculations have been made at the PBE+TS/HI level of theory.

**cation**	**silanols**	**$\Delta E_{ads}(CO_2)$/kJ mol^{-1}**	**$\Delta E_{disp}(CO_2)$ /kJ mol^{-1}**
K-CHA	(a) 6MR	-53	-27
Cs-CHA	(a) 6MR	-48	-26
K-CHA	(b) corner of 8MR	-59	-26
Cs-CHA	(b) corner of 8MR	-56	-32
K-CHA	(c) 8MR	-57	-27
Cs-CHA	(c) 8MR	-54	-29

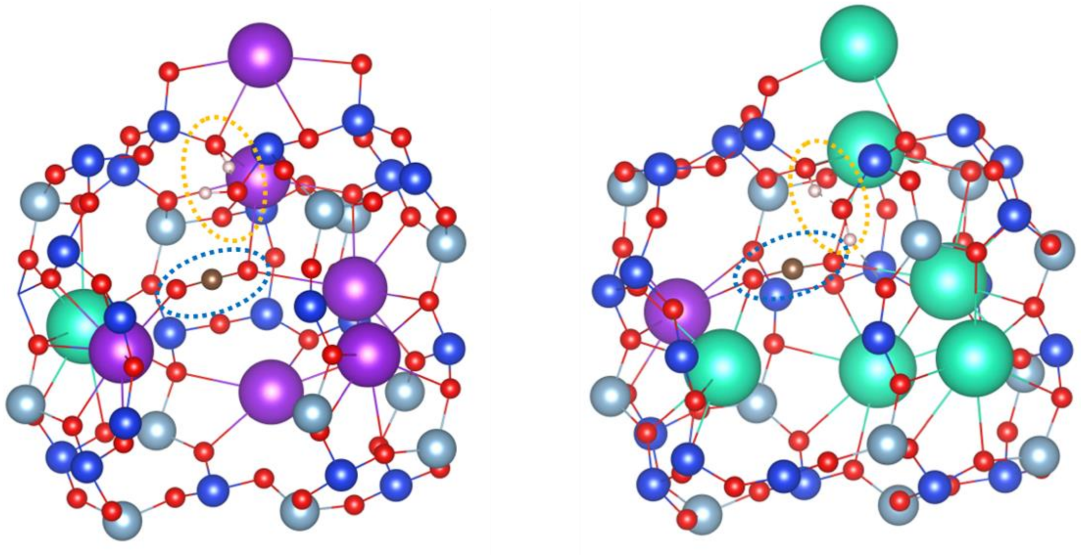

Fig. S6. Optimized structures after CO_2 adsorption with silanols in a 8MR (case c of Fig. 5 in the main text), for the model of K-CHA (left) and Cs-CHA (right). Color code: Si in blue, O in red, Al in grey, K in purple, Cs in green, and C in brown. Silanols are emphasized by a dashed orange line and CO_2 by a blue one.

Table S2. Computed chemical shifts of silanol protons, with and without adsorbed CO_2, with the tetramethylsilane (TMS) as a reference.

cation	silanols	δ ^{1}H (ppm), without CO_2		δ ^{1}H (ppm), with CO_2	
K-CHA	(a) 6MR	9.9	5.2	9.7	5.2
Cs-CHA	(a) 6MR	9.3	4.6	9.1	4.1
K-CHA	(b) corner of 8MR	6.9	8.5	7.6	8.5
Cs-CHA	(b) corner of 8MR	6.0	7.8	6.1	7.7
K-CHA	(c) 8MR	2.3	10.7	3.6	10.3
Cs-CHA	(c) 8MR	6.1	10.0	5.6	9.9

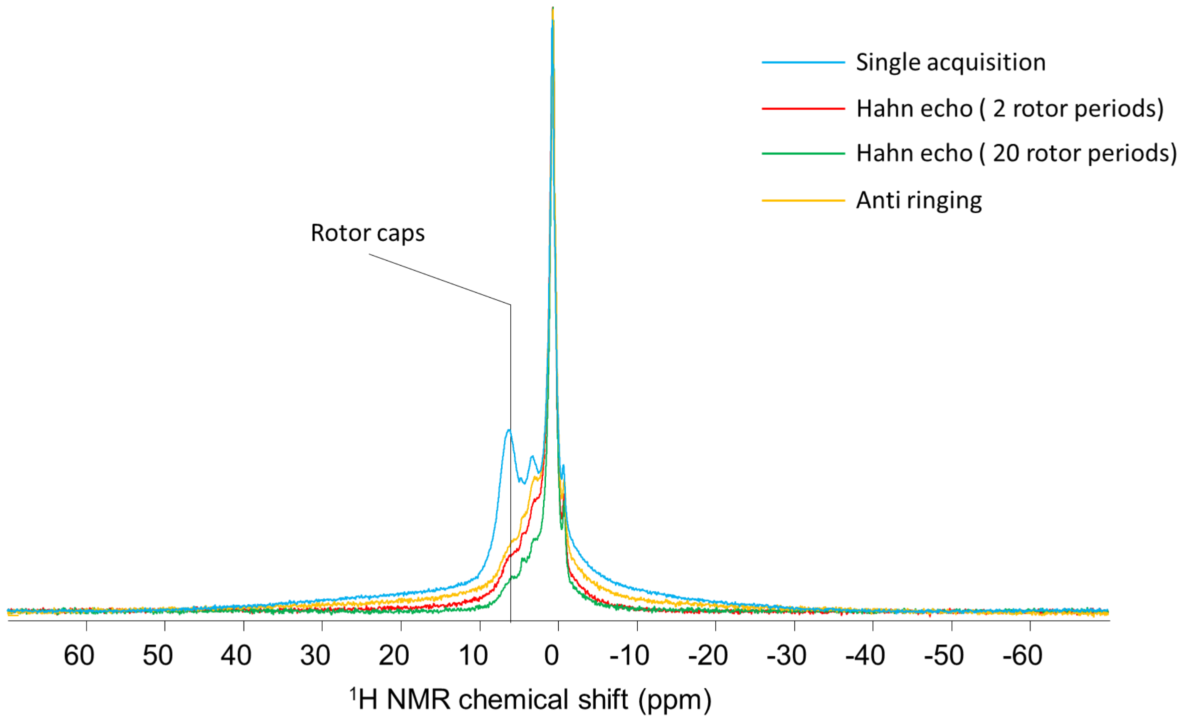

Fig. S7. ^{1}H NMR spectra of AP-CHA sample acquired at MAS frequency of 40 kHz using different approaches.

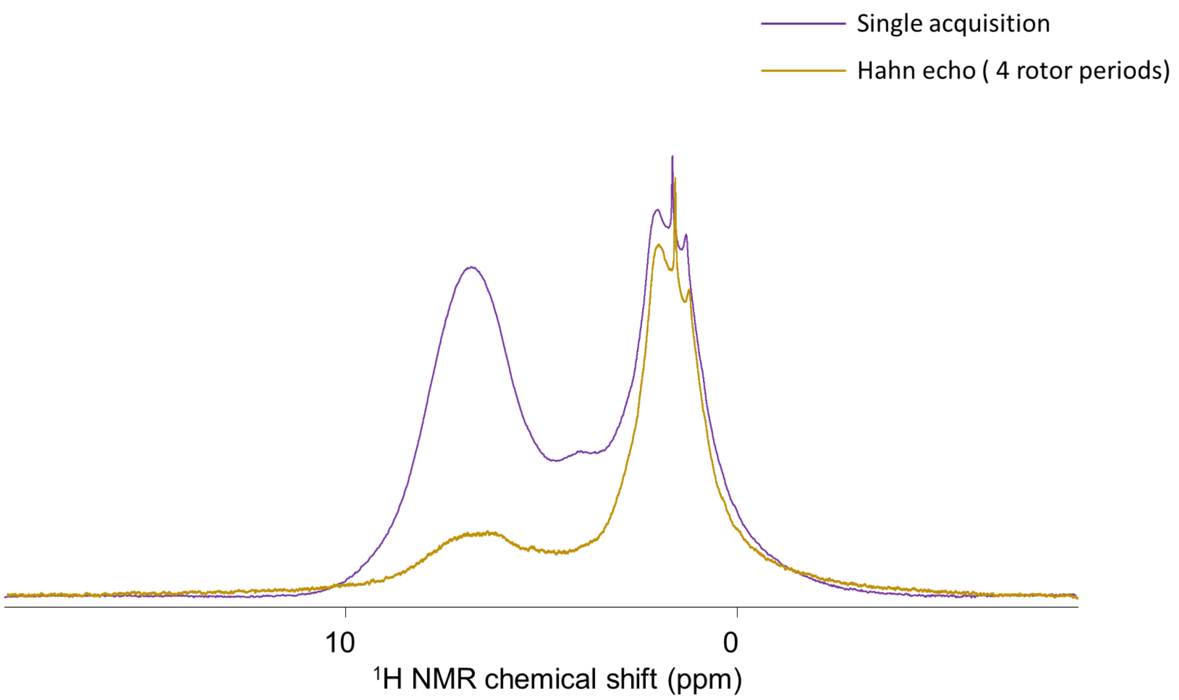

Fig. S8. ^{1}H NMR spectra of empty rotor acquired at MAS frequency of 40 kHz using different approaches.
